# Multilayer Solutions to Inequities During the COVID-19 Pandemic

**DOI:** 10.5888/pcd20.220354

**Published:** 2023-05-04

**Authors:** Krista Wonderly

**Affiliations:** 1 Wayne State University College of Nursing, Detroit, Michigan

At first glance, the Michigan Executive Directive No. 2020–7 is impressive and forward thinking. I initially lauded the executive directive that mandatory implicit bias training be required of all licensed health professionals. As stated in the order, “The COVID-19 pandemic has illustrated, with brutal proof, the persistence of racial disparities in our society . . . because of the prevalence of what is sometimes called *implicit bias*: thoughts and feelings that, by definition, often exist outside of conscious awareness, and therefore are difficult to control” (1). However, upon reading the directive in full, I noticed a theme that was important but too narrowly focused on me and my fellow health care professionals. It is not solely *our* bias in taking care of patients with COVID-19 that created the racial disparities or a surge in COVID-19–related deaths; interpersonal bias *and* structural implicit bias, in addition to discrimination, laid the foundation for the devastating statistics seen throughout Michigan and the United States.

As a critical care registered nurse in Detroit, Michigan, I was practicing in one of the epicenters of the pandemic and in the state with peak cases in March 2020. My coworkers and I take pride in providing excellent care to anyone who comes through our hospital doors, regardless of race or ethnicity. We have chosen to work for years in Detroit, whose demographics show that Black Americans comprise 78.3% of the population, while the overall population of Black Americans in the US is 12.8% (2,3). I agree that our “selfless and courageous service” was instrumental in preventing more lives from being lost (1). While it is undeniable that implicit bias has contributed to interpersonal bias that affects health outcomes, social determinants of health (SDOH) are also a part of why these patients were primarily at high risk for COVID-19.

SDOH, as defined by the Centers for Disease Control and Prevention (CDC), are the “wider set of forces and systems shaping the conditions of daily life that affect health outcomes” (4). Some examples of SDOH include safe housing, transportation, access to health care, environmental aspects such as polluted air and water, access to healthy food, options for physical activity, education, job opportunities, and many more. SDOH affect predisease conditions that increase risk of transmission of communicable diseases, conditions that increase risk of poor outcomes, and postdisease conditions that affect long-term outcomes (5). SDOH are key areas for research because, according to Behavioral Risk Factor Surveillance System data from 2017–2019, people who report experiencing 1 adverse SDOH have 1.6 increased odds of self-rated fair or poor health (6). The more social inequities one experiences, the greater the odds: those who report experiencing 4 or more adverse SDOH have 5.3 increased odds of self-reporting fair or poor health compared with those who report zero (6). In addition to reporting fair or poor physical health, those who experience 1 or more adverse SDOH have higher odds of reporting poor mental health days (6). The total burden of risk due to adverse SDOH is a significant predictor of health, beyond the influence of demographic characteristics alone (6).

Structural racism has contributed to the effects of SDOH and health inequity by reinforcing discriminatory beliefs in racial and ethnic minority populations. Historically, most studies have prioritized studying interpersonal racial and ethnic discrimination, with less focus on exploring the health effects of structural racism (7). Concentration on structural racism rather than interpersonal bias is crucial to improve health equity and ameliorate population health (7).

To address health outcomes further complicated by structural racism, a multilayer approach is needed among racial and ethnic minority populations. Racism is a structure — health care professionals can help to tear down this structure that contributes to health disparities, even if it must be demolished one brick at a time. While a multilayer method needs to address all SDOH, this essay highlights 2 contemporary conceptual models to provide a framework to advance future research in various health-related disciplines: the Assessing Community Engagement (ACE) Conceptual Model (8) and the housing and health disparities conceptual model (9).

## Housing Security

Care delivery bias was only one of many factors of structural and social determinants of health contributing to the racial and ethnic disparities during the COVID-19 pandemic in the US (5). Housing access is of concern for increased risk and risk of poor outcomes in the hospital and in the long term (5). The housing and health disparities conceptual model can be used to address health outcomes caused by structural inequalities through 4 pillars: cost, conditions, consistency, and context (9). Cost represents affordability, conditions encompass the adequacy of the physical environment, consistency describes residential stability and the ability of residents to remain in their home for as long as they wish, and context characterizes the surrounding health-relevant neighborhood resources (9). Mediating and moderating factors of structural inequality include differential vulnerability due to chronic stress, ability to acquire resources that promote health, differential vulnerability across the lifespan, and health behaviors that contribute to comorbid conditions such as smoking and lack of physical activity (9). When people are exposed to these factors, a multiplying cumulative exposure leads to poor health outcomes such as chronic and infectious disease. Addressing structural inequality and discrimination through cost, conditions, consistency, and context of housing can lead to improved health outcomes in, for example, chronic disease and maternal health (9). Disciplines including public health, nursing, social work, and medicine can implement this conceptual model to develop interventions in specific identified populations across all levels of health care. Additionally, screening tools based on these 4 pillars of housing equity need to be developed for use in hospital systems, outpatient clinics, and public health settings, allowing for increased awareness and connection to necessary social services and improved housing outcomes in patients served in that area. Addressing housing as a determinant of health equity can lead people to a healthier life.

## Community and Patient Engagement

The ACE Conceptual Model represents a guiding framework to use community engagement to drive the US toward health equity through systems modification (8). Community engagement is at the core of the conceptual model; changing health equity and systems can only happen through community engagement (8). According to this model, improving health care programs and policies requires that solutions come directly from the community (8). It is key that health care institutions, and health professionals working in those institutions, have a mutually shared goal surrounding the community’s needs. Medical mistrust may present itself further if the health care system implements changes in community health without having those crucial conversations. It is our job as health care professionals to listen to our patients and their families to hear what their needs are and to bring about that change within the health care system to serve the community at large. Once shared goals are identified, measurable actions should be taken to meet those goals, reassessed often, and adjusted if needed. Research is needed to identify tools to measure these goals and to develop implementation programs within neighborhoods. Moreover, intervention within community health should come from a place of true caring, instead of simply “checking off a box” for community engagement. To have a thriving community, measurable and attainable mutual goals must exist between health care systems and the communities they serve to achieve health equity through transformed systems of health.

## Conclusion

As a critical care nurse, I see some of the most acute patients in the hospital system, observing how their everyday lives have affected their health outcomes. As health care professionals, we must not forget that the patients and families we serve come from the community and then go back to the community once they leave us. No matter where we are in a person’s health care journey, it is our job to advocate for their health. The structural bias and racism that racial and ethnic minority patients endure every day is inexcusable. Health care professionals must address this issue by improving our own policies surrounding health equity. This essay has provided 2 conceptual frameworks with which to guide future research to address health inequities through housing and community engagement. Additionally, I encourage fellow health professionals to move forward with a larger conversation surrounding racial and ethnic minority health and share what we observe in our everyday practice to advance how we care for our patients.
